# Glutaredoxin in Rice Growth, Development, and Stress Resistance: Mechanisms and Research Advances

**DOI:** 10.3390/ijms242316968

**Published:** 2023-11-30

**Authors:** Rongrong Zhai, Shenghai Ye, Jing Ye, Mingming Wu, Guofu Zhu, Faming Yu, Xingyu Wang, Yue Feng, Xiaoming Zhang

**Affiliations:** 1 Institute of Crop and Nuclear Technology Utilization, Zhejiang Academy of Agricultural Sciences, Hangzhou 310021, China; 2 State Key Laboratory of Rice Biology, China National Rice Research Institute, Hangzhou 310006, China

**Keywords:** glutaredoxin (GRX), rice, growth development, stress resistance, breeding utilization

## Abstract

Rice (*Oryza sativa* L.) is a staple food for more than half of the global population. Various abiotic and biotic stresses lead to accumulation of reactive oxygen species in rice, which damage macromolecules and signaling pathways. Rice has evolved a variety of antioxidant systems, including glutaredoxin (GRX), that protect against various stressors. A total of 48 GRX gene loci have been identified on 11 of the 12 chromosomes of the rice genome; none were found on chromosome 9. GRX proteins were classified into four categories according to their active sites: CPYC, CGFS, CC, and GRL. In this paper, we summarized the recent research advances regarding the roles of GRX in rice development regulation and response to stresses, and discussed future research perspectives related to rice production. This review could provide information for rice researchers on the current status of the GRX and serve as guidance for breeding superior varieties.

## 1. Introduction

Rice (*Oryza sativa* L.) is a monocotyledonous model plant grown globally and a staple food for more than half of the global population [1]. The rice production and consumption in Asia accounts for approximately 90% of global production, and has an important role in the international economy [2].

The intensification of global climate change, abnormal temperatures, drought, salinity, and other abiotic stresses that occur frequently will have a huge impact on crop yields and food security worldwide within the next 20 years [3]. Under normal circumstances, the reactive oxygen species (ROS) content in plants is maintained at a low level, but under adverse conditions, such as low or high temperatures and drought, plants accumulate more ROS. It is generally accepted that low levels of ROS accumulation might be related to normal signal transduction pathways, whereas higher levels may affect the cellular redox state and cause oxidative damage [4].

ROS acted as important regulators in plant development in addition to oxidative stress responses. ROS was involved in the signaling of dormancy release and germination in seeds [5]. Emerging evidence suggests that ROS level and redox status change during anther development [6]. The role of hypoxia status in specifying anther cell identity was critical for subsequently triggering signal of ROS, inducing tapetal programmed cell death (PCD) to nourish microspores for producing mature pollen grains in rice, maize, and *Arabidopsis* [7].

Plant growth, development, and response to environmental stresses required the judicious dynamic balance of ROS [8]. To adapt to ROS accumulation caused by various stressors, plants have evolved a series of antioxidant systems, and the glutathione/glutaredoxin (GRX) system is a significant component of the redox pathway [9]. GRX regulates protein function by changing the intracellular redox state through switching between the SH- (thiol) and -S-S (disulfide) forms [8]. Electrons are first transferred from nicotinamide adenine dinucleotide phosphate to glutathione reductase, then to glutathione (GSH), and finally to GRX, which uses the acquired electrons to reduce S-S bonds between proteins [10,11]. 

Since GRX was first discovered in an *E. coli* mutant lacking the Trx gene [12], GRX genes have been partially characterized in *Oryza sativa* [13], *Arabidopsis thaliana* [14], and *Lycopersicon esculentum* [15], among others [16]. Recent reviews on plant GRX, in general, have comprehensively summarized the constantly accumulating knowledge within this field [8,17,18,19]. This review will focus specifically on the diversity and the function of rice GRX, summarize recent studies on the GRX involved in development regulation and responses to stress, and discuss their potential for genetic improvement in rice.

## 2. Diversity of GRX Genes in Rice

**Figure 1:**
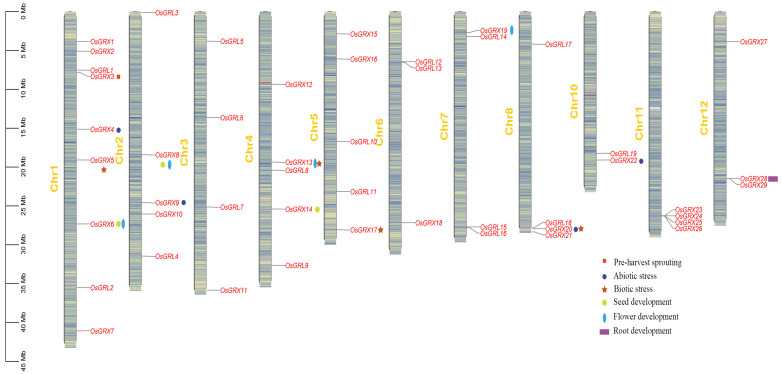
Physical map of glutaredoxin (GRX) genes in *Oryza sativa* L.

The 48 GRX gene names in rice were retrieved from the report by Garg et al. [20], and the genomic data were obtained from the rice genome annotation project website (http://rice.plantbiology.msu.edu/) (accessed on 25 May 2023). No GRX locus has yet been identified on chromosome 9, while nine, five, four, five, five, three, four, four, two, four, and three GRX loci mapped to chromosomes 1, 2, 3, 4, 5, 6, 7, 8, 10, 11, and 12, respectively. In addition, we visualized the chromosomal locations of GRX genes in rice [20] (Figure 1); this figure was adapted from Garg et al. [20]. Different numbers of GRX genes were found in clusters on chromosomes 11 and 12. Such gene clustering resulted from gene duplication, which might lead to functional redundancy or the creation of new functions [21].

**Figure 2:**
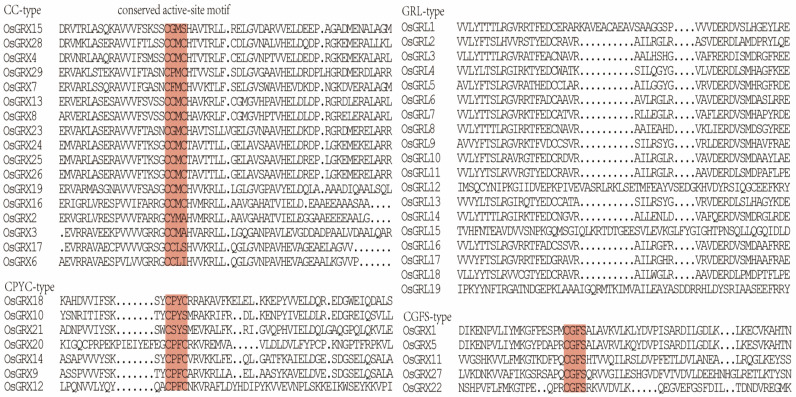
Multiple sequence alignment of representative GRX sequences from *Oryza sativa* L. Red shading indicates conserved GRX protein active sites.

**Figure 3:**
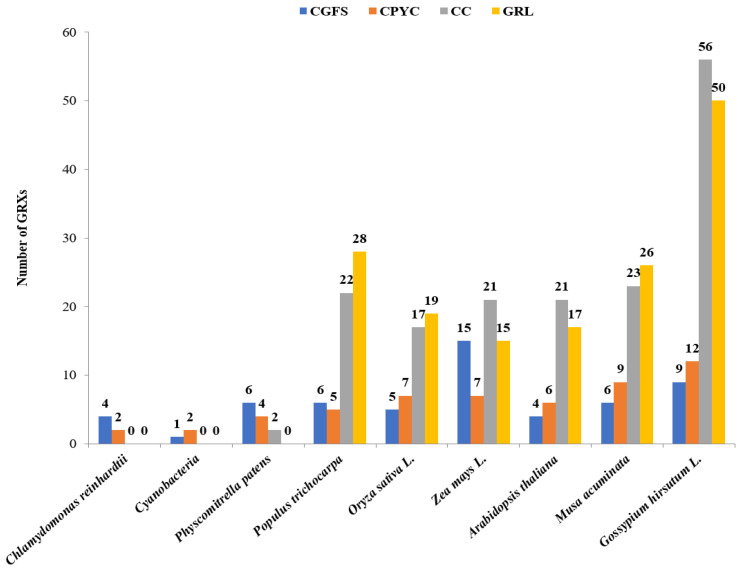
Distribution of GRX proteins in different subclasses.

Structurally, GRX proteins are small thiol-containing molecules of the thioredoxin-fold superfamily, which contains an active site with a specific sequence motif. In rice, GRX proteins were classified into four types, CPYC, CGFS, CC, and GRL, according to the conserved residues in their active sites (Figure 2) [20]. The active site of CPYC-type GRX proteins was C(P/S)(F/Y)(C/S), while that of CGFS-type GRX molecules was CGFS, which was structurally similar to CPYC-type sites. CGFS- and CPYC-type GRX proteins have been reported in a wide range of organisms, from prokaryotes to eukaryotes, while CC-type GRX motifs occurred only in higher plants [22]. The conserved CC-type GRX active site motifs extended to C(C/G/F/P/Y)(M/L)(A/C/I/S) in rice [23]. Members of the GRL-type class exhibited low homology to classical GRX proteins and do not harbor conserved active-site motifs [20]. There were 17 CC-type, 5 CGFS-type, 7 CPYC-type, and 19 GRL-type GRX molecules in rice [19]. Studies on numerous plants have shown that numbers of CC-type and GRL-type GRXs gradually increased during evolution (Figure 3), suggesting that these classes of GRX molecules might have roles in promoting the evolution of land plants with highly complex organs [24,25].

**Figure 4:**
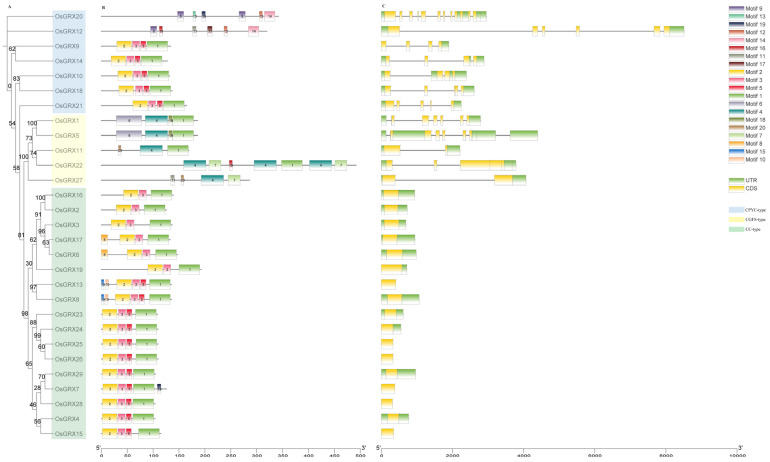
Phylogenetic relationships, gene structure, and motif compositions of CPYC-type, CGFS-type, and CC-type rice GRX genes. (**A**) Multiple alignment of 29 full length OsGRX proteins conducted using Clustal X 2.0 and a phylogenetic tree constructed using MEGA 11 with the neighbor-joining (NJ) method (5000 bootstrap replicates). (**B**) Schematic representation of conserved motifs in OsGRX proteins elucidated using TBtool v2.021. Each colored box represents a motif in the protein; motif names are indicated in the top right of boxes. (**C**) Structure of OsGRX genes coding sequences. Untranslated regions (UTRs), coding sequences (CDSs), and introns are represented by green boxes, yellow boxes, and lines, respectively.

To gain further insights into the possible evolution of protein structure and motif composition in OsGRX genes, we constructed a phylogenetic tree and analyzed the features of CC-type, CGFS-type, and CPYC-type GRX proteins (Figure 4A–C). As shown in Figure 4B, 20 distinct conserved regulatory motifs were predicted in CC-, CGFS-, and CPYC-type GRX proteins, with three to seven motifs in each protein. GRX proteins within each subfamily shared similar motif patterns, while motif compositions in CC- and CPYC-type subfamilies were also similar. We further analyzed OsGRX gene structures, as shown in Figure 4C, and the number of exons in OsGRX genes varied from one to twelve. Notably, all CC-type GRX genes were intronless. Such intronless gene classes/families evolved rapidly via gene duplication or reverse transcription, followed by integration into the genome, which might explain the large number of CC-type proteins detected [20,26].

## 3. GRX Proteins Participate in Rice Development Regulation and Response to Stresses

**Figure 5:**
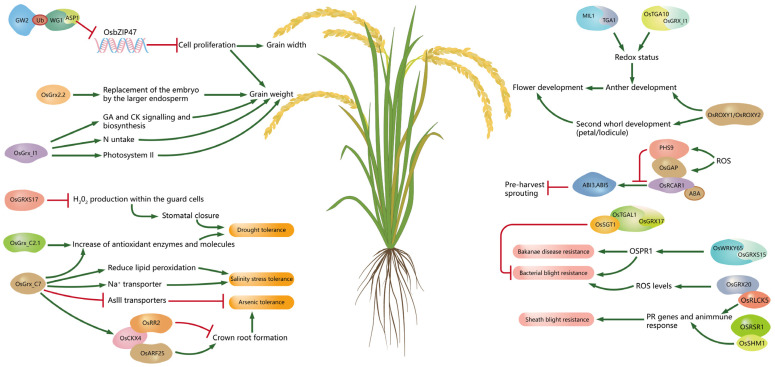
Mechanisms of GRX genes involved in rice growth, development, and stress resistance. Red and green lines indicate promotion and an inhibition, respectively.

GRX molecules have been extensively studied in humans and animals, while there have been relatively few studies in rice. To date, only eight of the seventeen CC-type, two of the five CGFS-type, and three of the seven CPYC-type GRX molecules have been characterized in rice (Table 1). GRX proteins were isolated from rice as early as 1997 [27], and subsequent studies have confirmed that they have roles in rice development and oxidative stress [28]. In the following sections, we describe some of the most important aspects of the involvement of GRX proteins in rice development regulation and response to stresses (Figure 5).

### 3.1. GRX Proteins Are Involved in Rice Seed Development

The rice grain size is mainly determined by the grain hull that limits grain growth, and several factors that control grain size by influencing cell proliferation and cell expansion in the grain hull have been reported in rice [46]. However, the molecular mechanisms of GRX proteins by which rice determines its seed size remain elusive. The TGACG-binding (TGA) transcription factor OsbZIP47 restricted grain growth by decreasing cell proliferation [37]. Hao et al. [37] found that WG1 (OsGRX8) acted as a conserved amino acid A(L/I)W(L/V) (ALWL) motif-containing adaptor protein that repressed OsbZIP47 transcriptional activity by recruiting the transcriptional co-repressor, ASP1. GW2, a functional E3 ubiquitin ligase, can control grain width and weight in rice by restricting cell proliferation in spikelet husks [47]. GW2 ubiquitinated WG1 and caused its degradation, thereby releasing inhibition of OsbZIP47 transcriptional activity. GW2-WG1-OsbZIP47 regulatory module controlled grain width and weight in rice [37]. An increase in grain weight was observed in *OsGRX6*-overexpressing plants [34]. *OsGRX6* also delayed rice plant senescence by slowing the degradation of chlorophyll and increasing the activity of photosystem II, thereby improving rice nutritional status [34].

With continued seed growth, most of the space in the mature seed is occupied by the endosperm in rice. Developmental coordination of the embryo and endosperm becomes crucial for the normal development of rice seeds. However, the associated molecular mechanisms remain poorly understood. *OsGrx2.2* (*OsGRX14*) regulated rice embryonic development, and its overexpression produced embryoless seeds, but seed weight was significantly increased, possibly due to replacement of the embryo by the larger endosperm [43]. *OsGRX14* was abundantly expressed in the aleurone layer of mature seeds and involved in seed tolerance to oxidative stress [5]. Furthermore, enhanced *OsGRX14* activity directly increased the ability of plants to clear ROS [5].

### 3.2. GRX Proteins Are Involved in Rice Flower Development

The development of male germ cells in flowering plants involves a series of complex biological events, including male meiosis, pollen development, and pollen maturation [7]. In rice, the ROS level and redox status changed at different cell specification stages during anther development [6]. ROS level was extremely low before stage 3, which is less than 200 pmol mg^−1^; the ROS level increased twice at stage 4 and stage 5 [6]. Redox homeostasis is important for specifying the cell identity of tapetal and microsporocyte cells [7]. CC-type GRX proteins regulated redox homeostasis and had crucial roles in flower development in rice [7].

Mutation of *MIL1* (*OsGRX19*) caused failure of secondary parietal cells to differentiate into the middle layer and tapetum cells in rice anthers [35]. MIL1 interacted with the TGA transcription factor, TGA1, eventually leading to GRX modification of Cys residues, thereby altering TGA1 transcriptional activity [39,48]. Mutation of *OsGRX_I1* (*OsGRX6*) led to programmed cell death of tapetal cells. Some researchers proposed that TGA1-MIL1 (OsGRX19) and OsTGA10-OsGRX_I1 (OsGRX6) had roles differentiated in time and space, with OsGRX6 potentially contributing to anther cell degeneration [7,35].

Mutants of *MIL1* (*OsGRX19*) gene homologs also caused defects in anther cell development [49]. *MSCA1*, a homolog of *MIL1* (*OsGRX19*) in maize, cooperated with the TGA transcription factor, FEA4, to control meristem size [50]. *ROXY1* and *ROXY2*, two *MIL1* gene homologs, controlled the initiation and differentiation of flower organs in *Arabidopsis*, where deficiency in either gene resulted in defective anthers and microspores [36]. Furthermore, ROXY1 and ROXY2 interacted with TGA9 and TGA10 during anther development, and mutations of *TGA9* or *TGA10* also caused anther cell developmental defects [51]. *OsROXY1* (*OsGRX13*) and *OsROXY2* (*OsGRX8*) in rice had more than 60% amino acid similarity with *Arabidopsis ROXY1*, and both proteins had CCMC-type active motifs [52]. *OsROXY1* (*OsGRX13*) and *OsROXY2* (*OsGRX8*) mediated petal and anther initiation and differentiation in *Arabidopsis* [36]. In situ hybridization showed that OsROXY1/OsROXY2 was expressed in the inflorescence meristem and briefly at the initial stage of flower organ development [36].

### 3.3. GRX Proteins Are Involved in Rice Root Development

The root system absorbs nutrients and water and is strongly associated with rice yield. Studies have shown that GRX proteins were involved in rice root development in rice [13,40]. Overexpression of *OsGRXC12* (*OsGRX28*) caused an obvious decrease in cortex and epidermis cell length in the lateral root apical differentiation zone, leading to lateral roots that were much shorter than those of wild-type controls [40]. Verma et al. [13] showed that *OsGrx_C7* (*OsGRX4*) promoted root growth and plant health by regulating the expression of the oxidative stress-induced root expansion-related genes, *OsMADS15*, *OsMADS25*, *OsWOX3*, *OsWOX11*, and *OsRR2.*

### 3.4. GRX Proteins Are Involved in Rice Pre-Harvest Sprouting (PHS)

PHS led to a loss of seed viability and reduced the yield and grain quality, and thus led to great economic loss [29]. ROS was demonstrated to play a signaling function in the alleviation of seed dormancy [53,54]. Abscisic acid (ABA) is an important hormone for the induction and maintenance of plant seed dormancy. Mutations of genes in this pathway may have caused plants to exhibit PHS [55]. However, the roles of integration of ROS signaling and ABA signaling in PHS in rice are far from understood. Unexpectedly, Xu et al. [29] identified a CC-type GRX protein, PHS9 (OsGRX3), and found that it links ABA signaling with the active oxygen signal via OsGAP, which interacts with the ABA receptor, OsRCAR1, thereby regulating pre-harvest rice germination. Expression of *PHS9* (*OsGRX3*) and *OsGAP* was promoted by H_2_O_2_ and suppressed ABA signaling, resulting in earlier germination.

### 3.5. GRX Proteins in Rice Responses to Abiotic Stress

#### 3.5.1. GRX Proteins in Rice Responses to Drought Stress

Drought directly affected soil microorganism and plant diversity, as well as negatively impacting other aspects of ecosystems. ROS production in cells was induced by drought, and ROS accumulation can disrupt the cellular redox balance [56]. Kumar et al. [33] overexpressed the rice GRX genes *OsGrx_C2.1* (*OsGRX9*) and *OsGrx_C7* (*OsGRX4*) in *Arabidopsis* and found that they significantly improved its resistance to drought stress. The authors speculated that this might be due to the fact that GRX proteins could reduce or eliminate the toxic effects of ROS production by promoting increased levels of antioxidant enzymes and molecules [30].

H_2_O_2_ is an important signal that regulates plant stomatal size, and high levels of H_2_O_2_ in guard cells help plants to withstand drought [57,58]. Rice plants with suppressed *OsGRXS17* (*OsGRX22*) exhibited elevated H_2_O_2_ production in the guard cells, increased sensitivity to ABA, and reduced stomatal apertures. Furthermore, silencing of *OsGRXS17* in rice can improve drought stress tolerance [42].

#### 3.5.2. GRX Proteins in Rice Responses to Salinity Stress

Salinity is a significant threat to the development and yield production of rice worldwide [59]. Salinity has been shown to affect total development by altering intricate interactions in nutrient absorption and accumulation, hormonal imbalance, and oxidative stress [60]. Oxidative stress disrupted cell redox balance, leading to ROS accumulation [61]. Plants have evolved a variety of adaptive systems to address the above challenges, among which GRX helps plants to cope with abiotic stresses [44]. *OsGrx_C7* (*OsGRX4*) expression was induced in rice under salinity stress, and plants over-expressing *OsGrx_C7* (*OsGRX4*) had a lower Na^+^/K^+^ ratio and lipid peroxidation levels, and higher proline and soluble sugar content. Furthermore, *OsGrx_C7* (*OsGRX4*) mediated salinity stress tolerance by increasing the content of proteins involved in Na^+^ transport [32].

#### 3.5.3. GRX Proteins in Rice Responses to Metal Stress

In plants, metal stress can lead to ROS production and accumulation, which in turn leads to DNA damage and non-specific oxidation of proteins and membrane lipids. As a highly toxic metal, arsenic toxicity directly affects plant growth and development [62]. To cope with the oxidative stress caused by arsenic, plants have evolved a variety of enzymes that can scavenge ROS, including GRX proteins, ascorbate peroxidase, catalase, and superoxide dismutase [63]. OsGRX genes can meditate arsenic detoxification in rice through glutathione recycling [30].

*OsGrx_C7* (*OsGRX4*) and *OsGrx_C2.1* reduced arsenic content, maintained the intracellular GSH pool, and improved arsenic tolerance in *Arabidopsis* [26]. *OsGrx_C7* also regulated the expression of arsenic III transporter genes (*OsNip1,1*, *OsNip3;1*, *OsLsi1*, and * OsLsi2*) in rice, thereby reducing arsenic III transport from roots to shoots, and ultimately reducing arsenic content in seeds [13]. Furthermore, overexpression of *OsGrx_C7* led to the production of a more extensive root system by affecting oxidative stress-induced root regulatory transcription factors (*OsMADS15*, *OsMADS25*, *OsWOX3*, and *OsWOX11*) and cytokinin-responsive root-related genes (*OsRR2* and *OsCKX4*), which could support increased accumulation of arsenic III in a bound form and reduce root to shoot arsenic III translocation [13]. Furthermore, the reduction in arsenic III in channel proteins facilitated higher nutrient flow, which could improve plant growth status [64].

### 3.6. GRX Proteins in Rice Responses to Biotic Stress

Bacterial blight and bakanae disease, caused by *Xanthomonas oryzae* pv. *oryzae* (*Xoo*) and *Fusarium fujikuroi*, respectively, are two serious bacterial diseases of rice. Rice sheath blight is a fungal disease caused by the soil-borne necrotrophic fungus, *Rhizoctonia solani* Kühn, which results in serious loss of rice yield. *Botrytis cinerea* is a typical necrotrophic pathogen that infects more than 200 plant species [65]. Limited numbers of OsGRX genes involved in disease resistance have been characterized.

TGA transcription factors OsTGAL1 negatively regulated resistance to *Xoo* by regulating the salicylic acid glucosyltransferase *OsSGT1* in rice [38]. Li et al. [38] identified a CC-type glutaredoxin, OsGRX17, which interacted with OsTGAL1. OsGRX17 decreased the ability of OsTGAL1 to bind to the *OsSGT1* promoter, thereby potentially influencing the OsTGAL1-mediated defense response to *Xoo*. OsGRXS15 (OsGRX5) interacted with the transcription factor, OsWRKY65, in the nucleus, and enhanced disease resistance to *Xoo* and *F. fujikuroi* by upregulating expression of *OsPR1*, which was related to pathogen responses [41]. *OsGRX20* positively regulated plant responses to bacterial and fungal attack. Overexpression of *OsGRX20* in rice significantly enhanced its resistance to bacterial blight attack and tolerance to methyl viologen and salt stress [44]. Wang et al. [45] showed that protein kinase domain-containing protein OsRLCK5 interacted with OsGRX20, which participated in the GSH-ascorbic acid antioxidant system, thereby regulating ROS balance to enhance rice resistance to sheath blight. The ectopic expression of the rice homolog, *OsROXY1*, in *Arabidopsis* led to increased H_2_O_2_ accumulation and enhanced susceptibility to *B. cinerea* [36].

## 4. GRX Proteins Cross-Talk with Hormones in Rice Development and Stress Responses

Hormones play an important role throughout the life cycle of plants, and changes in their synthesis or signaling pathways affect the morphology and development of plants. Studies have shown that the overexpression of *OsGRX6* in rice caused plants to become semi-dwarfs as it affected the metabolism of hormones, including gibberellin and cytokinin [34]. *OsGRX6* might alter the biosynthesis of gibberellin and cytokinin by increasing the expression of genes (*OsIPT-4*, *OsIPT7*, *OsIPT8*, *OsIPT9*, *OsIPT10*, *OsGA3ox2*, and *OsGA20ox2*) in the gibberellin and cytokinin pathways, thereby affecting the rice plant phenotype. Greenboim-Wainberg et al. [66] showed that *OsSPY*, a gibberellin negative regulatory gene, enhanced the cytokinin pathway in *Arabidopsis*. El-Kereamy et al. [34] further found that the overexpression of *OsGRX6* in rice led to the upregulation of *OsSPY* expression. Moreover, the increase in nitrogen content in rice shoots and seeds might be the result of increased cytokinin content by the overexpression of *OsGRX6* [34,67]. *PHS9* played an important role in the regulation of rice PHS through the integration of ROS signaling and ABA signaling [29].

Additionally, GRX could be affected by exogenous hormones. For example, *OsROXY2* (*OsGRX8*) was a homolog of *ROXY1*, and its transcription could be induced by exogenous auxin [68]. Sharma et al. [69] found that the overexpression of *OsGRX8* in *Arabidopsis* reduced its sensitivity to auxin and abscisic acid, and increased its tolerance to multiple abiotic stresses, including oxidative stress and salinity stress. The knockout of *OsGRX8* reduced the tolerance of *Arabidopsis* seedlings to the above-mentioned stresses. This might be due to the crosstalk between auxin and ROS signals, mediated by OsGRX8, thus regulating plant growth, development, and response to stress [69].

## 5. Perspectives

### 5.1. Prospects for Application of GRX Genes in Hybrid Rice Breeding

The breeding and large-scale adoption of hybrid seeds is an important achievement in agriculture. Cytoplasmic male sterile lines (CMS) and photoperiod/thermo-sensitive genic male sterile lines (PTGMS) are major sterile systems widely used in hybrid seed production; however, the efficiency of CMS resources for hybrid seed production is low, while PTGMS lines are often affected by environmental conditions. In contrast, genic male sterility (GMS) rice has advantages that can address the shortcomings of previous generations of hybrid rice technology.

Recessive nuclear male sterile genes insensitive to environmental conditions are widely distributed and ideal for hybrid rice breeding and production. In 2016, Chang et al. [70] constructed a male sterility system for hybrid rice breeding and seed production, using the nuclear male sterility gene, *OsNP1*. Zhen18B was the first GMS rice. Furthermore, a new technology based on Cas9 was established to develop a third-generation hybrid [71]. This strategy used the pollen fertility restoration gene, *CYP703A3*, and led to the generation of a maintainer, 9311-3B, with stable inheritance. With the availability of CRISPR/Cas9 technology and cloning of numerous GRX male sterile genes, maintainer lines centered on the male sterile rice genes, such as *MIL1* (*OsGRX19*) and *OsGRXI1* (*OsGRX6*), can be conveniently obtained to produce non-transgenic male sterile lines and hybrid seeds.

### 5.2. Prospects for Application of GRX Genes to Improve Rice Yields

Regulation of seed size is a key strategy for improving crop yield and has been a focus of much research to investigate the underlying mechanisms [72]. However, the molecular mechanisms by which plants determine their seed size remain elusive [37]. In particular, knowledge on the biological roles of members of the GRX family in seed development is still lacking. GW2, WG1/OsGRX8, and OsbZIP47 functioned in a common pathway to control grain growth by influencing cell proliferation. This mechanism differed from that of OsGRX6, which affected hormone signaling and nitrogen status in rice plants leading to increases in grain weight. Interestingly, however, both of the above two genes positively regulated grain weight. We believe that GRX molecules warrant further exploration in future studies to improve grain weight and yield using transgenic breeding approaches. It will be a challenge to exploit their natural alleles to increase grain size and weight in the future.

PHS in rice gives rise to a deterioration in the yield and quality of the grain, and thus results in great economic loss [73,74]. Severe PHS damaged about 6% of conventional rice and 20% of hybrid rice during the harvest season of southern China [75]. Numerous studies in rice have concluded that genes associated with PHS were mainly involved in ABA biosynthesis, catabolism, and signaling [76]. Xu et al. [29] identified a CC-type GRX molecule PHS9, which combined with OsGAP to disrupt ABA signaling and negatively regulated PHS in rice. Huaidao 5 and Wuyungeng 27 showed severe PHS in Zhejiang and Jiangsu province. Knockout of *PHS9* in Huaidao 5 and Wuyungeng 27 contributed to delayed germination by 2 days and had no effects on the phenotype of plant height, tiller number, grain shape, grain number per plant, grain chalkiness, and starch granules. These results demonstrated that *PHS9* could be a potential target for breeding PHS-resistant rice varieties using the CRISPR-Cas9 system [29].

### 5.3. Prospects for Application of GRX Genes in Alleviating Abiotic and Biotic Stresses

Rice (*Oryza sativa* L.), the world’s most consumed grain, is extremely sensitive to various abiotic and biotic stresses [77]. Abiotic stresses reportedly cause yield reductions up to 70% by adversely affecting rice survival, growth, and grain filling [78]. Similarly, biotic stresses such as pathogens (fungi and bacteria) impart severe yield losses or crop failure during infestation [79]. Approximately, 50% of rice yield was estimated to be lost due to bacterial blight disease worldwide [80]. Improving stress tolerance in rice is critical for increasing productivity to satisfy the projected food demands of the world population.

To date, molecular breeding and functional genomic studies have contributed to the understanding and improving growth and yield of rice under biotic and abiotic stresses [58,77]. As key stress-tolerance mediators, GRX could be manipulated to boost the tolerance to abiotic and biotic stresses in rice. In this review, we summarized recent reports about GRX in response to abiotic and biotic stresses in rice. Overexpressing *OsGRX4*, *OsGRX5*, *OsGRX20* or knockout *OsGRX17* or *OsGRX22* might result in improved stress tolerance. However, most tolerant rice materials obtained through introduction or knockout of single genes cannot be directly cultivated to generate tolerant varieties. To develop tolerant rice varieties, it will be necessary to introduce haplotypes comprising multiple genes involved in tolerance into rice varieties. On the other hand, based on the deep understanding of the genetic and molecular basis of crop domestication, de novo domestication of naturally resistant wild plants using gene-editing techniques may be a novel strategy to obtain resistant rice [81].

### 5.4. Prospects for Application of GRX Genes in Molecular Farming

Plant molecular farming uses plant organs or tissues as bioreactors for the production of recombinant proteins and bioactive metabolites via genetic engineering. Rice endosperm, as an ideal bioreactor, could be used to produce and store high-value active substances, such as pharmaceutical proteins, oral vaccines, vitamins, and nutraceuticals [82]. Overexpression of *OsGrx2.2* (*OsGRX14*) impaired embryo development, and subsequently led to increased endosperm size. The overexpressing plant may have the potential to become a high-value resource for producing more valuable nutrients and drugs. The *OsGrxC2.2*-overexpressed rice lines could be obtained by use of endosperm-specific promoters, such as *GluB4*, *GluD1*, *Gt-1*, *Gt-2*, *Gt-3*, and *Gt-13*, which were highly active and widely used for plant molecular farming [83,84].

## 6. Conclusions

Rice is widely cultivated worldwide as a major food crop, but its growth is affected by various abiotic and biotic stresses. Plants have evolved a series of antioxidant systems to reduce damage in response to the excessive ROS generated by abiotic and biotic stresses. GRX is a protein produced during plant evolution and is helpful for plants to cope with stresses. There are four main subtypes of GRX: CPYC, CGFS, CC, and GRL. To date, only eight of the seventeen CC-type GRXs, three of the five CGFC-type GRXs, and three of the seven CPYC-type GRXs have been characterized in rice. These genes are involved in rice seed development, flower development, root development, PHS, abiotic stress (drought, salinity, metal), and biotic stress (bacterial blight disease, bakanae disease, sheath blight disease). With the availability of CRISPR/Cas9 technology and other new biotechnologies, GRX genes can be applied in hybrid rice breeding, improving rice yields, alleviating abiotic and biotic stresses, and molecular farming. Understanding the mechanisms of GRXs could serve as guidance for breeding superior rice varieties.

## Figures and Tables

**Table 1:** Predicted and functionally characterized rice GRX genes.

Class	Chr.	Locus	Gene Name	Active Site	Functions	References
CC	1	*LOC_Os01g09830*	*OsGrx_A2/OsGRX2*	CYMA		
	1	*LOC_Os01g13950*	*OsGrx_A1/OsGRX3/PHS9*	CCMA	Pre-harvest sprouting	[29]
	1	*LOC_Os01g27140*	*OsGrx_C7/OsGRX4*	CCMC	Tolerance to arsenic, salinity, and drought stress	[13,30,31,32,33]
	1	*LOC_Os01g47760*	*OsGrx_I1/OsGRX6*	CCLI	Hormone and nitrogen status; flower development, grain weight	[34,35]
	1	*LOC_Os01g70990*	*OsGrx_C6/OsGRX7*	CFMC		
	2	*LOC_Os02g30850*	*OsGrx_C8/OsGRX8/* *OsROXY2/WG1*	CCMC	Flower development and pathogen response; grain size and weight	[36,37]
	4	*LOC_Os04g32300*	*OsGrx_C9/OsGRX13/OsROXY1*	CCMC	Flower development; response to pathogens	[36]
	5	*LOC_Os05g05730*	*OsGrx_S1/OsGRX15*	CGMS		
	5	*LOC_Os05g10930*	*OsGrx_C15/OsGRX16*	CCMC		
	5	*LOC_Os05g48930*	*OsGrx_S2/OsGRX17*	CCLS	Disease resistance to *Xoo*	[38]
	7	*LOC_Os07g05630*	*OsGrx_C10/OsGRX19/MIL1*	CCMC	Anther development and microspore formation	[39]
	11	*LOC_Os11g43520*	*OsGrx_C17/OsGRX23*	CGMC		
	11	*LOC_Os11g43530*	*OsGrx_C13/OsGRX24*	CCMC		
	11	*LOC_Os11g43550*	*OsGrx_C14/OsGRX25*	CCMC		
	11	*LOC_Os11g43580*	*OsGrx_C16/OsGRX26*	CCMC		
	12	*LOC_Os12g35330*	*OsGrx_C12/OsGRX28*	CCMC	Lateral root elongation	[40]
	12	*LOC_Os12g35340*	*OsGrx_C11/OsGRX29*	CPMC		
CGFS	1	*LOC_Os01g07950*	*OsGrx_S15/OsGRX1*	CGFS		
	1	*LOC_Os01g34620*	*OsGrx_S15.1/OsGRX5/OsGRXS15*	CGFS	Disease resistance to *Xoo* and *Fusariumfujikuroi*	[41]
	3	*LOC_Os03g63420*	*OsGrx_S14/OsGRX11*	CGFS		
	10	*LOC_Os10g35720*	*OsGrx_S17/OsGRX22/OsGRXS17*	CGFS	Tolerance to drought stress	[42]
	12	*LOC_Os12g07650*	*OsGrx_S16/OsGRX27*	CGFS		
CPYC	2	*LOC_Os02g40500*	*OsGrx_C2.1/OsGRX9*	CPFC	Tolerance to arsenic and drought stress	[31,33]
	2	*LOC_Os02g43180*	*OsGrx_C3/OsGRX10*	CPYS		
	4	*LOC_Os04g17050*	*OsGRX12*	CPFC		
	4	*LOC_Os04g42930*	*OsGrx_C2.2/OsGRX14*	CPFC	Embryo development and grain weight; oxidative stress in developing and mature seeds	[5,43]
	6	*LOC_Os06g44910*	*OsGrx_C4/OsGRX18*	CPYC		
		*LOC_Os08g44400*	*OsGRX20*	CPFC	Tolerance to salt, cold, and heat stresses; resistance to sheath blight	[44,45]
	8	*LOC_Os08g45140*	*OsGrx_S12/OsGRX21*	CSYS		
GRL	1	*LOC_Os01g13480*	*OsGRL1*			
	1	*LOC_Os01g61350*	*OsGRL2*			
	2	*LOC_Os02g01200*	*OsGRL3*			
	2	*LOC_Os02g51370*	*OsGRL4*			
	3	*LOC_Os03g07470*	*OsGRL5*			
	3	*LOC_Os03g24030*	*OsGRL6*			
	3	*LOC_Os03g44650*	*OsGRL7*			
	3	*LOC_Os04g33680*	*OsGRL8*			
	4	*LOC_Os04g54860*	*OsGRL9*			
	4	*LOC_Os05g28530*	*OsGRL10*			
	5	*LOC_Os05g39450*	*OsGRL11*			
	5	*LOC_Os06g12030*	*OsGRL12*			
	6	*LOC_Os06g12190*	*OsGRL13*			
	6	*LOC_Os07g06600*	*OsGRL14*			
	7	*LOC_Os07g46410*	*OsGRL15*			
	7	*LOC_Os07g46570*	*OsGRL16*			
	8	*LOC_Os08g07450*	*OsGRL17*			
	10	*LOC_Os08g44070*	*OsGRL18*			
	12	*LOC_Os10g34170*	*OsGRL19*			
